# Obesity and hypertensive heart disease: focus on body composition and sex differences

**DOI:** 10.1186/s13098-016-0193-x

**Published:** 2016-11-30

**Authors:** Giovanni de Simone, Costantino Mancusi, Raffaele Izzo, Maria Angela Losi, L. Aldo Ferrara

**Affiliations:** 1 Hypertension Research Center, Federico II University Hospital, Via S. Pansini 5, Building 1, 80131 Naples, Italy; 2 Department of Translational Medical Sciences, Federico II University Hospital, Naples, Italy; 3 Department of Advanced Medical Bioscience, Federico II University Hospital, Naples, Italy

**Keywords:** Obesity, Hypertension, Left ventricular mass, Body composition

## Abstract

There is evidence that hypertension is frequently associated with overweight/obesity even in kids and adolescents. Either conditions influence development of left ventricular (LV) hypertrophy (LVH), through different biological and hemodynamic mechanisms: obesity is conventionally thought to elicit a coherent growth of LV chamber dimensions and myocardial wall thickness (eccentric LV geometry), whereas a more accentuated increase in wall-thickness (concentric LV geometry) is attributed to hypertension. While during youth these differences are visible, proportion of LV concentric geometry, the most harmful LV geometric pattern, sharply raises in obese individuals during middle age, and becomes the most frequent geometric patterns among obese-hypertensive individuals. Two conditions with elevated hemodynamic impact, severe obstructive sleep apnea and masked hypertension contribute to the development of such a geometric pattern, but non-hemodynamic factors, and specifically body composition, also influence prevalence of concentric LV geometry. Contrasting a general belief, it has been observed that adipose mass strongly influences LV mass, particularly in women, especially when fat-free mass is relatively deficient. Thus, though blood pressure control is mandatory for prevention and reduction of LVH in obese hypertensive patients, without reduction of visceral adiposity regression of LVH is difficult. Future researches should be addressed on (1) assessing whether LVH resulting from alteration of body composition carries the same prognosis as pressure overload LVH; (2) defining tissue characterization of the hypertrophic heart in obese-hypertensive patients; (3) evaluating whether assessment of hemodynamic loading conditions and biological markers can help defining management of the association of obesity with hypertension.

## Background

**Fig. 1:**
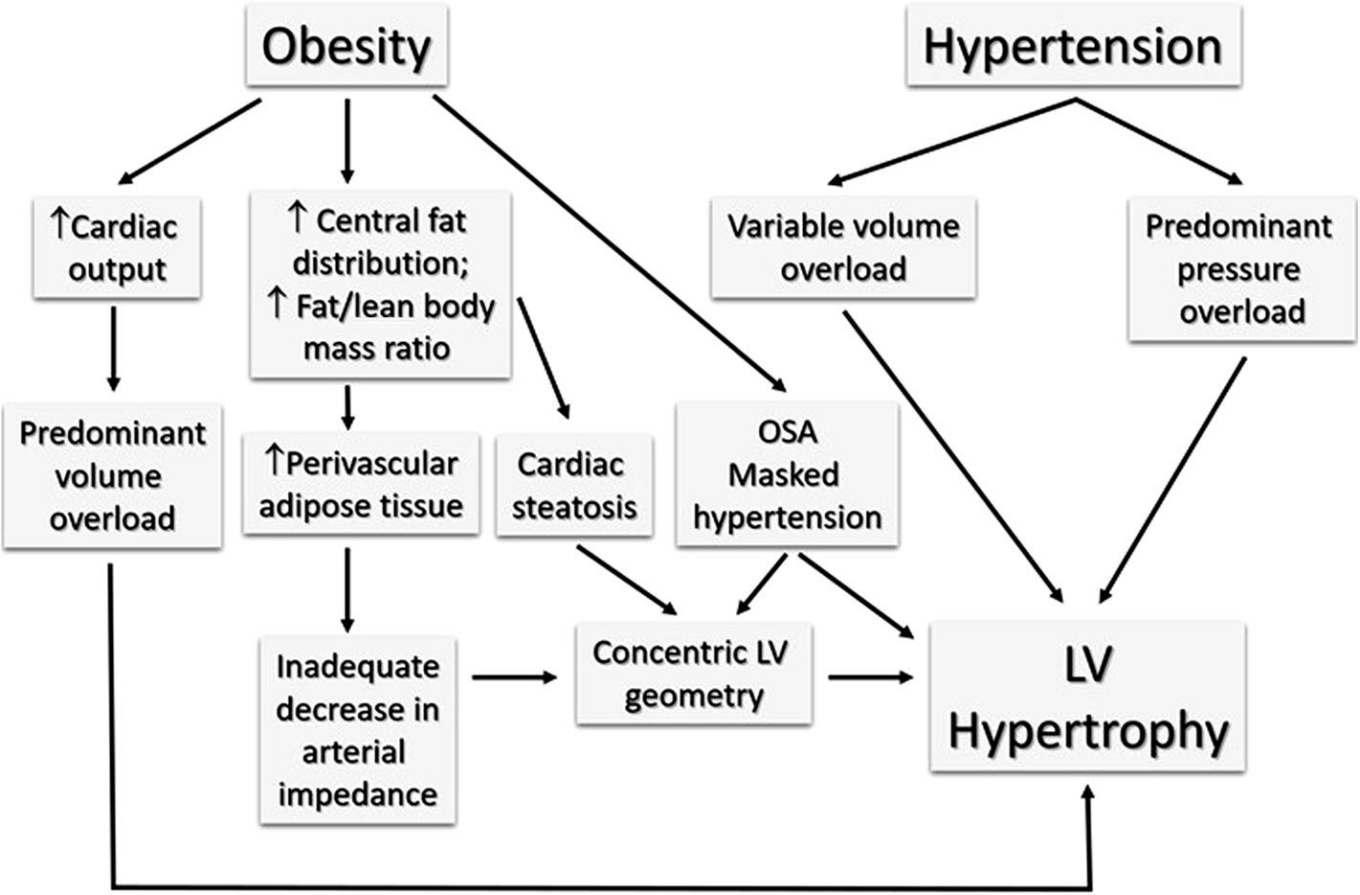
Physiologic changes promoting common cardiovascular modifications in obesity and hypertension. *OSA* obstructive sleep apnea, *LV* left ventricular

In this mini-review we will examine the impact of obesity in the hypertensive population, focusing on the common cardiovascular changes and interaction, leading to considerations on sex difference and body composition and potential influence on management (Fig. 1).

## Obesity as a comorbidity in arterial hypertension

More than 80% of hypertensive patients present with additional risk factors, including glucose intolerance, hyperinsulinemia, lipid disorders (reduced HDL-cholesterol and increased LDL-cholesterol and triglycerides) and obesity [1–4]. More than 50% of hypertensive patients present at least 2 of these comorbidities and, generally, one of those is obesity [4].

There is strong evidence that the prevalence of hypertension increases sharply with increasing body weight [2]. Scattered longitudinal analyses also suggest that obesity is an important risk factor also for incident hypertension, especially in women [5, 6].

Recently, the Southern Community Cohort Study, a large population-based survey, reported the probability of hypertension rising from 56% in overweight participants, to 2.5 fold in class I/II obesity, and up to 4.5 times in morbid obese participants [7]. This sharp progression of probability is also evident in kids and adolescents [8–10], though a significant decline might be observed in the past 10 years [11].

In a Caribbean population based study [10], among 2023 5-to-16 year-old public school students, the prevalence of hypertension was 13% in normal-weight individuals, raising to 23% in overweight and as much as 53% in obese subjects.

An important methodological issue, when assessing blood pressure in the obese patient, is the cuff size. Blood pressure measurement must take into account upper-arm circumference and the cuff size in both office and home setting [12]. Use of small cuff size causes overestimation of blood pressure and often increases the incidence of white coat or masked hypertension [13].

## Cardiovascular modifications of obesity

Evaluation of obesity-related cardiovascular changes poses method problems, especially when normalization for body size is needed. The traditional use of body surface area (BSA) is inappropriate, because of geometric inconsistencies between BSA and LV mass, but also because body weight (and therefor fat mass) is included in the computation of BSA [14]. Thus using BSA produces a substantial underestimation of the prevalence of LV hypertrophy (LVH) in population with high prevalence of obesity [15]. Using of allometric signal of body height, obtained in a reference population [14] and recently confirmed in a different cohort study [16], nearly doubles the population risk attributable to LVH, especially when prevalence of obesity is high [17].

Observation of cardiovascular modifications of obesity during adolescence are particularly helpful to highlight the consistency of abnormalities of obesity with those found in the context of arterial hypertension.

**Fig. 2:**
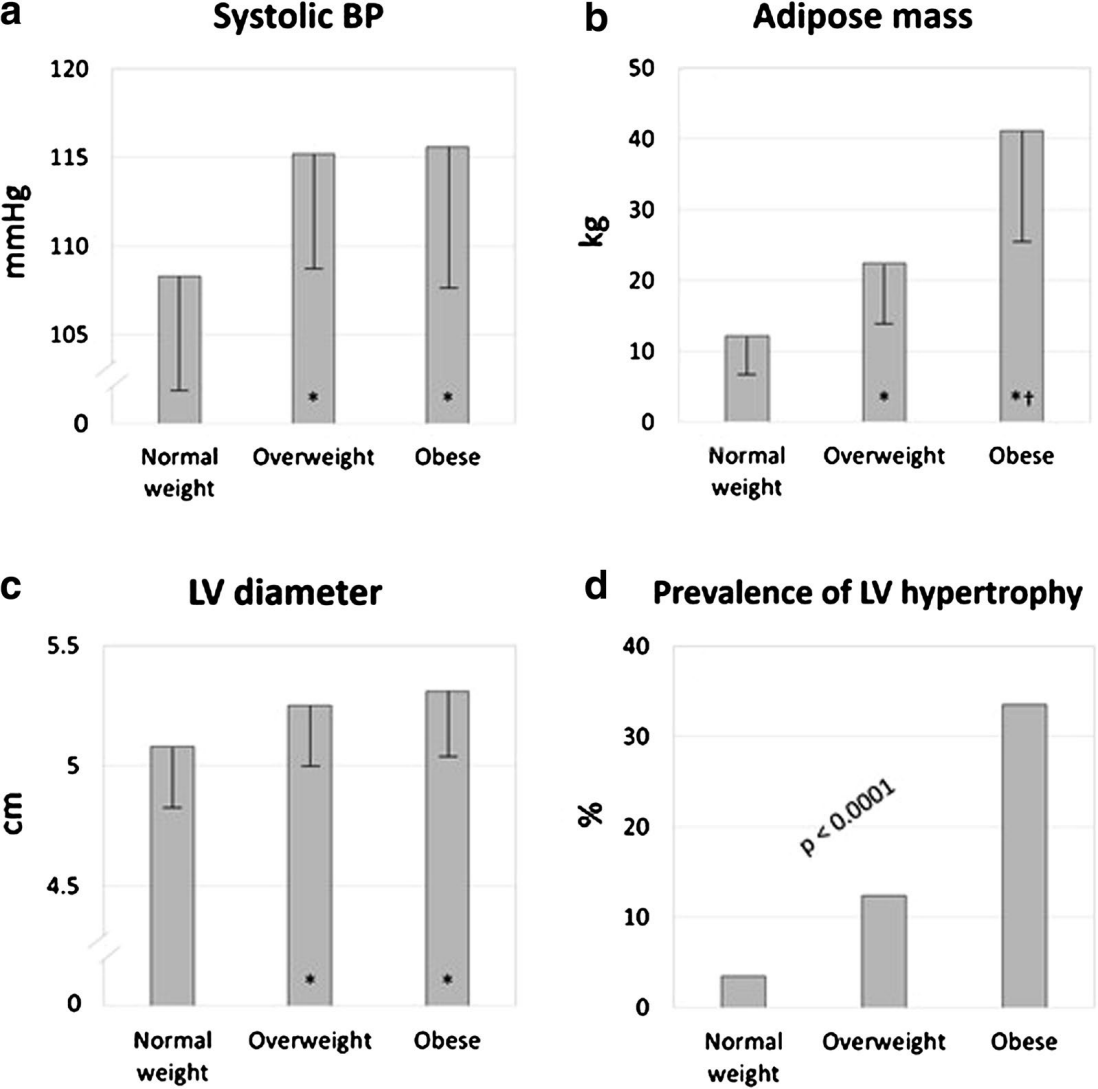
Systolic blood pressure (BP, **a**), adipose mass (**b**), left ventricular (LV) diastolic diameter (**c**) and prevalence of LV hypertrophy (**d**) in normal weight (n = 114), overweight (n = 113) and obese adolescents (n = 223). Note that prevalence of LV hypertrophy follows the excess of adipose mass, more than BP or LV chamber dimension. Adapted from Ref. 12. *Asterisk* significant vs normal weight subgroup; *dagger* significant vs overweight subgroup

In the young obese offspring participants of the Strong Heart Family Study cohort [18], although prevalence of occasional high BP was low (10% in the obese sub-population), clear-cut LV hypertrophy (LVH) was progressively greater in overweight and obese adolescents compared to normal-weight individuals, paralleling the magnitude of adipose mass (Fig. 2). LV geometry was mainly eccentric [18]. In the HyperGEN registry [19], the impact of obesity, diabetes and dyslipidemia (labeled generically as ≪metabolic risk factors≫) were assessed relatively to the probability of LVH in both normotensive and hypertensive subjects. The probability of LVH in normotensive participants with 1 or more metabolic risk factors was as high as the probability of LVH in hypertensive subjects without any of them, and was independently related to central fat distribution and associated ≪metabolic risk factors≫. The association of metabolic risk factors with hypertension increases remarkably the probability of LVH.

The proportion of LV concentric geometry, the most deleterious LV geometric pattern [20], sharply raises during middle age [21, 22] and becomes the most frequent LV geometric patterns among obese individuals [23, 24]. Thus, the association of obesity with hypertension produces a combined pressure and volume overload, which yields specific LV geometric abnormalities [20] and the most severe degrees of LVH, similar to that reported for the hemodynamic overload caused by specific valvular heart disease producing combined volume and pressure overload [19] [25].

The traditional paradigm of cardiovascular modifications in obesity with hypertension has been that obese individuals mostly develop eccentric LVH, due to the increased volume overload [26, 27]. However, this paradigm, is changing [28], because of the increasing evidence that concentric LV geometry is very prevalent in obese individuals [21, 22, 24, 29]. In a population sample of hypertensive New York employees [23], the combination of hypertension and obesity was associated with a greater prevalence of concentric LVH than in normal-weight participants. Woodiwiss et al. [29] studied a population sample of untreated hypertensive patients and found that obesity promotes LV concentric remodeling and hypertrophy rather than eccentric LVH. Similarly and more recently, Turkbey et al. [24] found that concentric LVH was the most frequent LV geometric pattern in obesity, using magnetic resonance imaging in the large population of the MESA study, forcing one to consider revising the prevalent viewpoint [28].

In obese patient, the increase in relative wall thickness (the most used index of concentricity) and LV mass is also typically associated with evidence of subclinical LV systolic dysfunction, especially when hypertension coexists, such as depressed midwall shortening, abnormal reduced tissue myocardial velocities, strain and strain rate [30, 31]. In the presence of concentric LV geometry, ejection fraction is an imprecise marker of systolic function [32], because the cardiomyocyte contraction is amplified across the wall thickness by the interplay of cross-fiber thickening and shortening of differently aligned muscular fibers [33, 34].

Many mechanisms have been proposed to explain the association of obesity with hypertensive target organ damage, including enhanced sympathetic system activity, production of inflammatory cytokines and endothelial dysfunction [35–37]. These mechanisms are related to the circulatory and tissue conditions associated with obesity.

## Abnormal conditions associated with cardiovascular modifications in obesity

### Hemodynamic load

Two conditions are frequent in obesity, potentially helping explaining the prevalent concentric LV geometry: severe obstructive sleep apnea (OSA) and masked hypertension.

OSA has been demonstrated to elicit concentric LV geometry [38], substantially due to the frequent pressure overload due to the phases of apnea and the consequent drop of O_2_ saturation [39]. OSA also causes a chronic mild inflammatory status, a characteristic frequently reported in the context of central obesity [40].

Another stimulus to continuous pressure overload is masked hypertension, reported to be more frequent in obesity [41]. The reasons of this association is not completely clear. Cardiac output is greater in obesity, in resting conditions, and daily activities might further increase output to a level that is not compensated by adequate decrease in both pulsatile and steady components of arterial impedance, to maintain normal blood pressure [42], mainly due to obesity associated endothelial dysfunction [43–45]. This abnormal adaptation generates a pressure overload superimposed to the “natural” volume overload pattern seen in obesity. Advanced glycation end-products and peri-vascular fat might contribute to the mismatch between flow output and vascular capacitance [46, 47].

In addition to the hemodynamic changes that help explaining prevalence of concentric LV geometry, there is also some evidence that increased sarcomere production is not the sole phenomenon to be involved in the increased myocardial mass occurring in obesity [48].

### Body composition

A paradigm in the pathophysiology of obesity is that the increased LV mass is a function of the increased fat-free mass, a typical feature of increased body weight also among obese individuals [49, 50], whereas adipose mass has little influence [51, 52]. However, body composition has an important impact on LV geometry in hypertensive-obese individuals. When the visceral component of adipose mass is considered, a close correlation with LV mass becomes evident [53, 54], with differences between men and women.

**Fig. 3:**
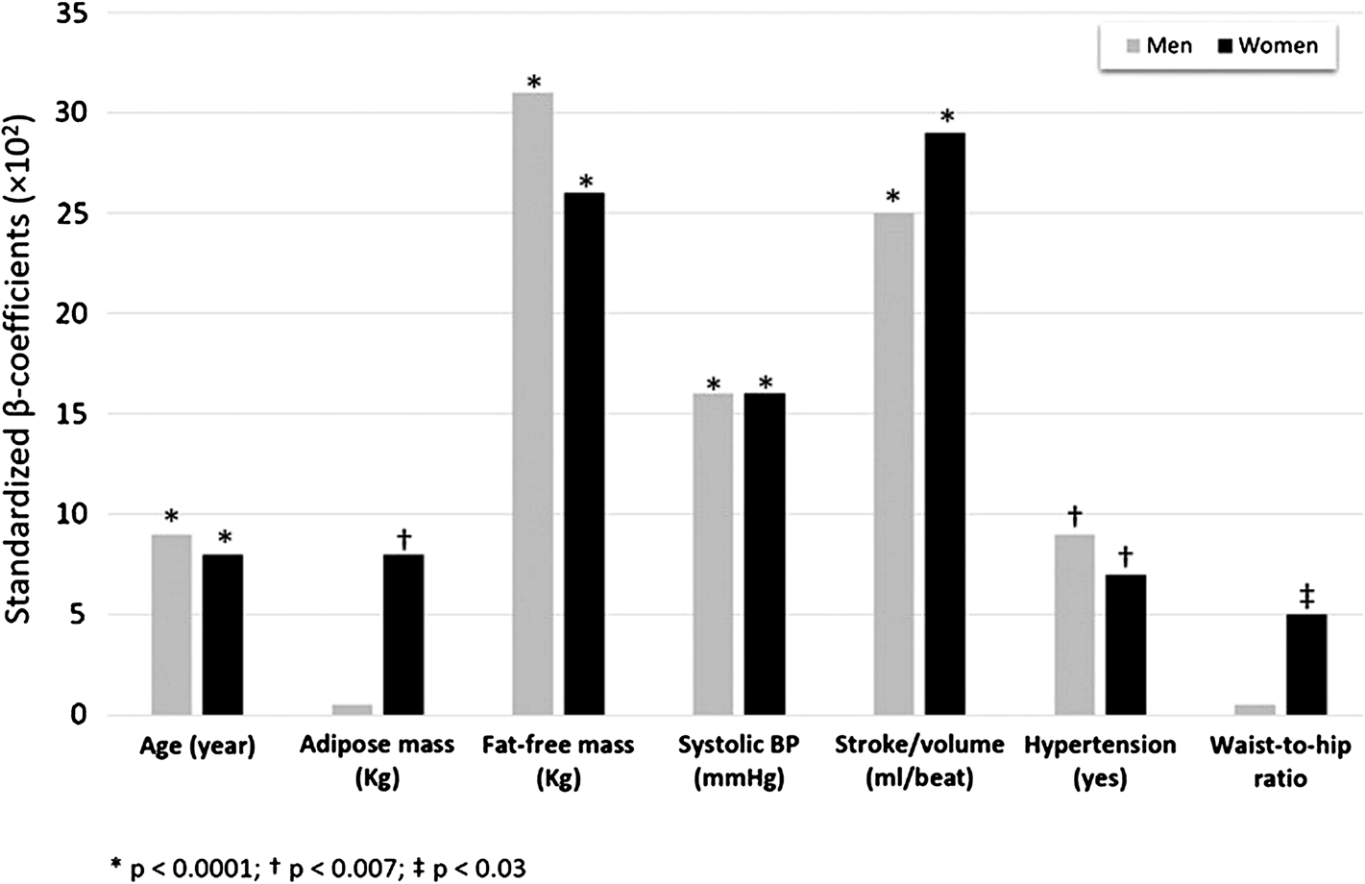
Relative contribution of parameters associated with variance of LV mass index, by standardized β-coefficients obtained by multiple linear regression analysis in the cohort of the Strong Heart Study. Adipose mass together with waist-to-hip ratio exhibit significant association with LV mass index variance only in women. From Ref. [50]

On epidemiological scale, in women, the importance of adipose mass on variability of LV mass is as high as that of arterial hypertension [50], whereas fat-free mass is independently related to LV mass in both men and women. The “cardiac steatosis” also has functional consequences in women but not in men [55]. The statistical effect of adipose mass on variability of LV mass in women is interrelated to the visceral distribution, as indicated by the significant impact of waist-to-hip ratio (Fig. 3).

Adipose mass also influences LV geometry. In a clinical study involving lean, obese and former-obese individuals [56], LV relative wall thickness, a potent measure of concentric LV geometry, was closely related to adipose mass (mostly intra-abdominal), and not to lean body mass. This relation was even closer than the relation of relative wall thickness with systolic BP. Based on these findings, one might postulate that visceral fat contributes by non-hemodynamic pathways to the development of concentric LV geometry in obese individuals. If this postulate holds truth, one may speculate that in obese subjects with severe abnormalities of body composition (i.e. with excess body fat/fat-free mass deficiency), LVH and LV concentric geometry should be even more severe.

## Adipose mass and LV geometry

We specifically studied the effect of fat mass in a special sub-population of obese individuals exhibiting a relative deficiency of fat-free mass and a consequent excess of adipose mass [54], a condition that is sometimes defined as ≪sarcopenic obesity≫ [57]. The identification of this condition is not easy because in obesity fat-free mass is always increased in absolute terms [49, 50, 58]. In the strong heart study cohort, a relative fat-free mass deficiency was found in 46% of the obese population, a characteristic that was substantially more frequent in women than in men. According to the old paradigm of LV mass only related to fat-free mass, lower LV mass could be expected in obese individuals with relative fat-free mass deficiency. In contrast, though formed by the 90% of women, this relatively ≪sarcopenic≫ sub-group exhibited larger LV chamber size and greater LV mass (even when normalizing for fat-free mass) with a clear trend toward concentric LV geometry. This peculiar LV geometric pattern was associated with values of blood pressure similar to the subgroup of obese subjects with normal fat-free mass, the same prevalence of diagnosed arterial hypertension, but higher inflammatory markers. It is clear that in this context, the increased LV mass cannot be considered as a pure consequence of biological mechanisms induced by the hemodynamic load.

There is a further demonstration that hypertensive LVH developing in obesity is unmatched with hemodynamic loading condition. When LV geometric adaptation is evaluated in relation to the need to sustain hemodynamic load, obesity is associated with excess of LV mass, in both men and women, relative to the magnitude of myocardial mass that would be adequate to sustain the individual hemodynamic load [50]. Women, in whom sarcopenia is much more prevalent, exhibit levels of excess of LV mass substantially greater than men, strongly suggesting, therefore, that there might be alterations in the normal myocardial structure associated with the increased LV mass. The complex of these findings forces to reconsider the old Virkow’s definition of ≪the fatty heart≫ [59].

There is strong evidence that overnutrition produces adipocyte infiltration in the liver, pancreas, kidney, muscles and heart, and triglycerides accumulate into the cells [60]. At the heart level, this has been directly demonstrated using cine-magnetic resonance imaging and a technique called ≪localized proton spectroscopy≫ , able to identify exactly the triglyceride peak. The amount of triglycerides in the myocardium is closely related to BMI and has been directly demonstrated in specimens using oil red O staining [55, 56, 61]. Thus, the measure of LV mass in obese individuals includes cell populations and cell components that are different from what measured in lean subjects. The increased amount of fat infiltrating the myocardium carries 2 important functional consequences, the overwhelming availability of fatty acids and the consequent increase in insulin resistance [62, 63]. The consequence of this biological alteration is a switch in the energy production, with the further reduction of carbohydrate oxidation and the overwhelming fatty acids oxidation which is a less efficient way to produce energy [64]. The reduced myocardial mechano-energetic efficiency related to fatty acid oxidation carries negative effects in terms of cardiovascular outcome and is substantially more prevalent in obesity [65].

Thus, the increased LV mass in obesity is related not only to the increased muscle component, which is elicited by the increased hemodynamic load. The expansion of myocardial tissue related to non-muscular components (adipocyte infiltration, fibrosis, cardiomyocyte thickening) [60, 61] helps understanding the tendency to more prevalent concentric LV geometry, as the ratio between fat and fat-free mass increases. Under this scenario, the concentric LV geometry related to obesity would not be a consequence of cardiomyocyte thickening due to parallel superimposition of sarcomeres, but rather infiltration of non-muscular components [48]. Superimposing arterial hypertension to this unfavorable structural pattern produces the greatest levels of LVH because of both hemodynamic (combined pressure and volume overload) and non-hemodynamic reasons (fat infiltration, inflammation).

## Blood pressure control in the obese patient

In general, the hemodynamic pattern typical of the combination of obesity and hypertension is characterized by increased intravascular volume component that should be taken into account when managing arterial hypertension [20].

Controlling blood pressure is difficult in the presence of obesity. In the Campania Salute Network, the probability to optimally control blood pressure decreases with increasing BMI, despite the greater number of medications used [66, 67]. The frequency of increased BP despite aggressive therapy in the context of obesity is likely to be due to mechanisms related to the autocrine and paracrine activity of perivascular fat, namely the production of angiotensin II and aldosterone, making peculiar the origin of hypertension in obese individuals [68]. We also postulate that studying the characteristics of the hemodynamic load might help understanding a more pathophysiologic way to manage arterial hypertension in the presence of obesity, based on the combination between types of hemodynamic overload and metabolic abnormalities, as we have recently proposed [22].

**Fig. 4:**
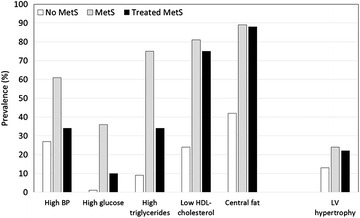
Prevalence of single components of metabolic syndrome (MetS), according to the NCEP-ATPIII definition, in the participants in the HyprGEN study (from Ref. 47). *Columns* represents the prevalence in the sub-population without MetS (*white*) or with present MetS (*grey*) or with diagnosis of MetS, partially controlled by treatment of single components. Prevalence of all risk factors is significantly reduced in the managed MetS sub-group (0.05 < p < 0.0001), except central fat. LV hypertrophy is not influenced by the control of those risk factors, including hypertension

Even in the presence of good control of BP, not necessarily LV mass follows the decrease in BP when obesity is present. In the HyperGEN registry, partial correction of metabolic abnormalities and high BP characterizing metabolic syndrome, but without reduction of central obesity, did not result in a corresponding lower LV mass [69], suggesting that a consistent reduction of LV mass cannot be achieved in this context without a program of weight reduction (Fig. 4).

## Weight loss and regression of cardiovascular abnormalities

Dietary intervention and bariatric surgery are the mainstay for obesity treatment. Abnormal LV geometry and function can substantially improve after weight loss in both adults and adolescents. Weight loss is associated with reduction of LV hypertrophy in obese individuals [70, 71], an effect that is even more evident, compared with the effect of blood pressure reduction on reduction of LV mass [49, 72]. In observational studies of high-risk hypertensive patients, a given decrease in blood pressure is less effective to reduce LV mass when obesity is present [73].

An extreme option is bariatric surgery, which is very effective to control the cluster of obesity-related cardiovascular risk factors, hypertension and diabetes (or insulin-resistance) [74]. In adults with morbid obesity, weight loss after bariatric surgery is associated with improvement of LV systolic function and normalization of LV geometry also due to the consistent reduction of systolic BP [75, 76]. After bariatric surgery, even adolescent obese subjects exhibit regression of LV hypertrophy and a trend toward normalization of LV concentric geometry [77].

## Current recommendations and need for research

LVH is the most relevant marker of cardiovascular risk in arterial hypertension and current guidelines strongly recommend decrease LV mass. However, given the particularity of LVH in the combination of arterial hypertension with obesity, whether or not LVH has the same prognostic impact in both obese and non-obese hypertensive individuals is still unclear.

Current guidelines for arterial hypertension [78, 79] do not have specific suggestions for treatment of arterial hypertension and regression of LVH in the presence of obesity. Also, the recent recommendations for the management of obesity [80, 81], while strongly suggesting the study of body composition and highlighting the peculiarities of the combination of obesity with hypertension, do not have clear indication on management.

We think that efforts should be oriented to characterize:

1. Whether LVH has the same pathophysiological meaning and, therefore, prognostic impact in both obese and non-obese men and women;
2. The tissue characterization of the hypertrophic heart in obese-hypertensive patients, a possibility emerging from new technologies applied to nuclear magnetic resonance [82, 83];
3. Whether extensive assessment of hemodynamic loading conditions and biological markers can help defining management of the association of obesity with hypertension even beyond the crude intervention on loading conditions.

## Conclusions

Obesity and hypertension are closely linked conditions. Obesity exaggerates LV response to increased hemodynamic load in large part throughout the effect of non-hemodynamic mechanisms elicited by visceral adiposity. Fat mass is as important as, and perhaps more important than, fat-free mass to promote increase in LV mass in visceral obesity, which is especially evident in women, related to their body composition. Body composition is likely to be a key factor in determining LV geometry in the presence of obesity and is at the basis of the paradoxical sex-difference found in LV adaptation to arterial hypertension. Effectively controlling BP and reducing hypertensive LVH is difficult without managing obesity. Research should be implemented to find more adequate approach to manage this frequent combination.
